# Assessing the potential of partial root zone drying and mulching for improving the productivity of cotton under arid climate

**DOI:** 10.1007/s11356-021-15259-6

**Published:** 2021-07-30

**Authors:** Rashid Iqbal, Muhammad Habib-ur-Rahman, Muhammad Aown Sammar Raza, Muhammad Waqas, Rao Muhammad Ikram, Muhammad Zeshan Ahmed, Monika Toleikiene, Muhammad Ayaz, Farhan Mustafa, Salman Ahmad, Muhammad Usman Aslam, Muhammad Mohsin Waqas, Muhammad Tahir Khan, Muhammad Mahran Aslam, Imran Haider

**Affiliations:** 1 Department of Agronomy, Faculty of Agriculture & Environment, The Islamia University of Bahawalpur, Bahawalpur, Pakistan; 2 Department of Environmental Sciences, University of Okara, Okara, Pakistan; 3 Institute of Crop Science and Resource Conservation (INRES) Crop Science Group, University Bonn, Bonn, Germany; 4 Department of Agronomy, MNS-University of Agriculture, Multan, Pakistan; 5 Institute of Plant Protection, MNS-University of Agriculture, Multan, Pakistan; 6 Lithuanian Center for Agriculture and Forestry (LAMMC), Kėdainių, Lithuania; 7 Collaborative Innovation Center on Forecast and Evaluation of Meteorological Disasters, Key Laboratory for Aerosol-Cloud-Precipitation of China Meteorological Administration, Key Laboratory of Meteorological Disasters, Ministry of Education, Nanjing University of Information Science and Technology, Nanjing, 210044 China; 8 Department of Agricultural Engineering, Khwaja Fareed University of Engineering & Information Technology, Rahim Yar Khan, Pakistan; 9 Nuclear Institute of Agriculture (NIA), Tando Jam, 70060 Pakistan

**Keywords:** Antioxidant enzyme activities;, Abscisic acid;, Gas exchange;, Irrigation;, Water use efficiency

## Abstract

Water scarcity constrains global cotton production. However, partial root-zone drying (PRD) and mulching can be used as good techniques to save water and enhance crop production, especially in arid regions. This study aimed to evaluate the effects of mulching for water conservation in an arid environment under PRD and to further assess the osmotic adjustment and enzymatic activities for sustainable cotton production. The study was carried out for 2 years in field conditions using mulches (NM = no mulch, BPM = black plastic mulch at 32 kg ha^-1^, WSM = wheat straw mulch at 3 tons ha^-1^, CSM = cotton sticks mulch at 10 tons ha^-1^) and two irrigation levels (FI = full irrigation and PRD (50% less water than FI). High seed cotton yield (SCY) achieved in FI+WSM (4457 and 4248 kg ha^-1^ in 2017 and 2018, respectively) and even in PRD+WSM followed by BPM>CSM>NM under FI and PRD for both years. The higher SCY and traits observed in FI+WSM and PRD+WSM compared with the others were attributed to the improved water use efficiency and gaseous exchange traits, increased hormone production (ABA), osmolyte accumulation, and enhanced antioxidants to scavenge the excess reactive oxygen. Furthermore, better cotton quality traits were also observed under WSM either with FI or PRD irrigation regimes. Mulches applications found effective to control the weeds in the order as BPM>WSM>CSM. In general, PRD can be used as an effective stratagem to save moisture along with WSM, which ultimately can improve cotton yield in the water-scarce regions under arid climatic regions. It may prove as a good adaptation strategy under current and future water shortage scenarios of climate change.

## Introduction

Cotton (*Gossypium hirsutum* L.) is a fiber crop, which boosts the economy of various countries around the world. Cotton is the second major crop in Pakistan after wheat. Its production declined due to various factors; among environmental issues is the most contributing factor (GOP, 2016-2017; Rahman et al. 2016). One of the main climatic issues that can affect agricultural production negatively is drought. Many field crops including cotton require a high amount of water and extra irrigation for successful production under arid climatic conditions. Currently, both climate change and environmental issues resulted in a shortage of agricultural water for crops (Rahman et al. 2020; Salehnia et al. 2020; Arshad et al. 2021). Therefore, increasing water use efficiency (WUE) requires more emphasis on crop-water management strategies considering crop physiology especially in water-scarce arid regions.

Farming practices consume around 70% of the global freshwater from the existing water resources in the world (Sepaskhah and Ahmadi 2012) and as well as in Pakistan. Global freshwater resources are reducing sharply. Therefore, adaptation and developing new irrigation techniques are necessary for efficient use of irrigation water (Kang and Zhang 2004; Iqbal and Raza 2019; Ghaffar et al. 2020) to combat water-scarce scenarios under current and future climate change especially in Pakistan (Rahman et al. 2018; Saddique et al. 2020). The partial root-zone drying irrigation technique (PRD) is one of the modern approaches of deficit irrigation technique with dividing a crop root-zone into two parts. The technique essentially involves irrigating approximately half of the root system of a crop while the other half is left to dry. Following a certain period, the dry half of the root system is irrigated, while the previously irrigated half is left to dry (Adu et al. 2018; Iqbal et al. 2021). In this way, the dry half gives hormonal signals, and then the hormones are carried through the xylem vessels to the plant shoots resulting in a partial closure of stomata that reduces the vegetative growth, transpiration rate, and water use of the whole plant (Stoll et al. 2000). Irrigated half at the same time provides water to the shoots, avoiding plant water deficiency and maintaining plant water status in the plant shoots (Dry and Loveys 2000; Adu et al. 2018).

Recent studies on PRD irrigation technique for wheat (Iqbal and Raza 2019; Ahmad et al. 2020) cotton (Iqbal et al. 2020) had shown significant benefits including conservation of plant water in association with the reduction of soil evaporation losses and increase in the irrigation efficiency (Marsal et al. 2008). The PRD has also shown some direct impacts on crop growth and development as fruit quality improvement in grapes (Du et al. 2008, b) changes in accumulation and composition of anthocyanin (Bindon et al. 2008). Drought stress or water scarcity stimulates the production of reactive oxygen species (ROS) that can cause damage to any structure or organelles of plant cells. These ROSs can make very fast chemical bonds to alter the nature of lipids/fats, which are in contact with them. The protective enzymes that are produced under water stress can be helpful to cut down detrimental effects and employed in the defense mechanism of plants such as ascorbate peroxidase (APX), catalase (CAT), peroxidase (POD), and superoxide dismutase (SOD), which are helpful for ROS scavenging and plant turgor maintenance (Shareef and Gui, 2018; Raza et al. 2017; Shareef et al. 2018a, Iqbal et al. 2020).

The other way for soil moisture conservation and improving fertility is mulch application (Nalayini et al. 2009; Ahmad et al. 2019; Iqbal et al. 2020; Zou et al. 2021). Mulch has a wide range of application materials and performs a range of functions for both soil and plant such as improvement of the soil infiltration rate, cutting down water runoff, decreasing the evaporation losses, and reducing weed growth (Ahmad et al. 2015; Ahmad et al. 2020; Iqbal et al. 2020; Ahmad et al. 2021; Khan et al. 2021; Perveen et al. 2021). In water-scarce regions with low water inputs, mulch is among the best solution for optimum plants growth and development (Iqbal et al. 2020; Ahmad et al. 2020), because it enhances soil organic matter and moisture content for proper root growth and therefore increases water holding capacity (Khurshid et al. 2006). Straw mulches are more beneficial in comparison with black plastic and cotton sticks (Ahmad et al. 2020; Ghosh et al. 2006). Scientists used various types of mulches (plastic, straws) for tomato production and concluded that the highest tomato production was resulted from applying the wheat straw mulch (Arin and Ankara, 2001). Other studies also confirmed that wheat straw performed better amongst all the straw mulches, for the production of various crops (Grassbaugh et al. 2002; Sanchez et al. 2008; Kosterna, 2014).

Since cotton is the main fiber and cash crop usually grown in water-limited areas and is commonly irrigated in an artificial way consuming a plenty amount of water, PRD is among the best way of irrigation without causing a major reduction in crop growth and yield (Tang et al. 2005; Gu et al. 2004; Rahman et al. 2019; Mehboob et al. 2020). Although the PRD itself is among the best techniques for water conservation, combining it with other methods for water conservation such as mulch application might increase water use efficiency by saving water from being evaporated.

To our knowledge, limited studies have considered a combination of the PRD irrigation technique and the mulch application for the production of cotton under arid environmental conditions. Therefore, to provide farmers and agricultural stakeholders with the necessary information for best adaptation strategies for water use efficiency, research should address the combined effects of PRD and mulch application for cotton production. The objectives of study were (i) to evaluate the positive and negative effects of mulching for water conservation in arid conditions under PRD and (ii) to assess the osmotic adjustment and enzymatic activities under two irrigation methods of FI and PRD considering with and without mulch conditions for the better cotton production under arid climatic regions

## Materials and methods

### Experimental and crop management details

Experiments were carried out using two irrigation techniques viz. full irrigation (control) and PRD with the combination of various mulches (NM = no mulch/bare soil, BPM = black plastic mulch at 32kg ha^-1^, WSM = wheat straw mulch at 3 tons ha^-1^, CSM = cotton sticks mulch at 10 tons ha^-1^) at a research area of Islamia University (Bahawalpur, Pakistan) for the period of 2017–2018. The study area is located in arid climatic conditions, where cotton crop faced high extreme temperatures during reproductive growth phases. High average temperature was recorded in 2018 as monthly temperature ranges from 32.35 to 34.2 °C (May to October) than 2017 (29.5 to 30.9 °C). To maintain the efficacy of PRD, a moving rain-out shelter was used to avoid rainfall moisture. Climatic information during both study years is presented in Table 1.

**Table 1:** Growing season (monthly) climatic data of experimental site for 2017 and 2018

Years	Month	T_max_ (^0^C)	T_min_ (^0^C)	T_average_ (^0^C)	RH (%)	Rainfall (mm)
2017	May	38.4	23.4	30.9	66.4	-
	June	44.9	27.3	36.1	60.2	0.4
	July	48.7	31.1	40.0	67.3	-
	August	45.3	33.2	39.2	68.4	0.6
	September	40.8	24.6	32.7	70.5	-
	October	36.2	22.9	29.5	72.1	0.8
2018	May	39.1	29.3	34.2	62.2	0.3
	June	45.3	34.2	39.7	58.3	-
	July	52.4	36.4	44.4	55.2	-
	August	44.3	33.7	39.0	67.3	0.2
	September	43.2	31.2	37.2	66.1	-
	October	38.4	26.3	32.3	73.4	-

*T*_*max*_, maximum temperature; *T*_*min*_, minimum temperature; *T*_*average*_, average temperature; *RH*, relative humidity

Experiments were arranged in a randomized complete block design (RCBD) with a split plot arrangement, having three replications, keeping the irrigation regimes in main plots and mulches in sub-plots. Cotton cultivar, MM-58 (drought tolerant) obtained from Regional Agricultural Research Institute (RARI), Bahawalpur, is widely being cultivated at farmer fields in the region. Thereafter, the seeds were sown on beds by keeping a plot size of 11m × 22m experimental unit. To avoid water movement between the designed treatments, an area of 5 m was left between each plot. Phenological data about the cotton growth stages are presented in Table 2 during both cotton-growing years.

**Table 2:** Cotton phenological events days after sowing (DAS) for both growing year

Phenology	Year 1 (2017)		Year 2 (2018)
Sowing	6 May		12 May
Emergence	11		15
First node on main stem	18		22
Tenth node on main stem	46		49
Squaring	56		59
First bloom	71		75
Full bloom	86		92
First open boll	93		100
Early boll loading	107		114
Mid boll loading	121		128
Mature bolls (50% open bolls*)	135		142
Boll Dehiscence	142		156
First Harvest	146 (28 September 2017)		158 (18 October 2018)
Second Harvest	163 (15 October 2017)		167 (27 October 2018)

^*^Last irrigation

**Fig. 1:**
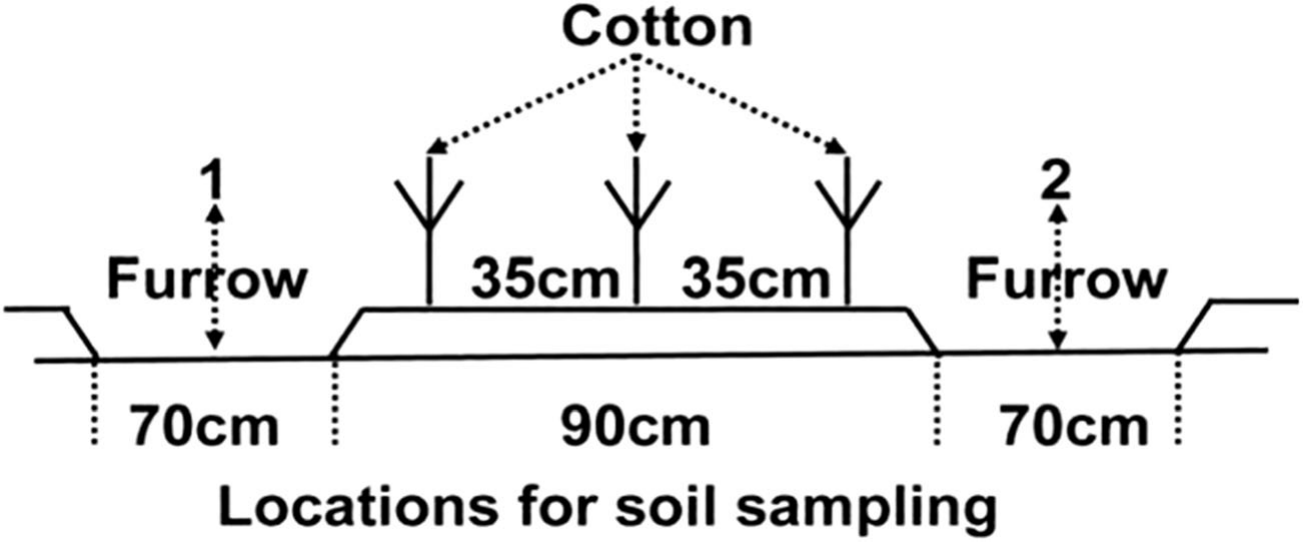
Soil sampling locations under field conditions for water monitoring, crop rows, and furrows cross-sectional perpendicular front view. 1 and 2 indicate alternate (wet & dry) furrows (70cm width) for irrigation and mulches application. Cotton seeds were sown on the bed (35cm between the rows and 10cm within the row). Mulches were spread in furrows and between the rows

Each sowing bed consists of three rows including 35 cm distances between the rows and 10 cm within the rows. Three cotton rows (on the bed) were established between two furrows with a width of 70 cm for each furrow as shown in Fig. 1.

All the plants were irrigated equally until the emergence of the crop and after that, various mulches were applied in furrows and between the rows. After 15 days of sowing, water was applied as full irrigation for control treatment according to the crop requirement, while 50% less water was applied in partial root-zone drying treatments. Moreover, the amount of each irrigation is given in Table 3.

**Table 3:** Irrigation scheduling and amount (mm) for cotton plants under field conditions for the year 2017 and 2018

Sowing (S) and Irrigations (I)	Years		Irrigation Amounts (mm)	
	2017	2018		
S	6 May	12 May	FI	PRD
I	22 April	28 April	150	150
I	20 May	26 May	160	80
I	25 June	01 July	160	80
I	09 July	15 July	170	85
I	29 July	05 August	160	80
I	20 August	28 August	150	75
I	20 September	29 September	100	50
Total			1050	686

Note: FI and PRD are full irrigation and partial rootzone drying irrigation, respectively. Half amount of water is used in PRD experimental plots. Water was applied to the furrows using pipes and the water amount was controlled by flow meters

Mulches were applied after emergence and removed before the last irrigation from the field sub-plots. Soil temperature was not recorded because there was no physical damage to crop plants due to the low intensity of mulches used. The diammonium phosphate (DAP) was used at the rate of 500 kg ha^-1^ and mixed in the upper 25cm soil before sowing. Urea was applied in June during both years at 350 kg ha^-1^ in furrows that were irrigated later on. Chemical sprays were not used against weeds due to the aim of evaluation/control of weeds density and biomass under PRD and FI using with and without mulches.

The timing and amount of irrigation for FI followed according to the local commercial practice. Irrigation was supplied when the sign of mid-day leaf wilting reached up to 50% using plastic pipes (12 cm in diameter). A flow meter at discharging end of the pipe was installed to calculate the amount of water. The timing of irrigation for PRD also the same as for FI but 50% less water was used in PRD. The PRD and FI were applied after calculating the water requirement of cotton. Total water calculation at the end of the experiment in control treatment was calculated as 1050 mm with and without mulch application and 686 mm for PRD as an alternate to irrigation for both with and without mulch application. Irrigation amount was applied with time presented in Table 3 during the cotton-growing seasons (2017 and 2018).

### Measurements and calculations at 10–12 a.m.

All the physiological measurements and sampling were taken on the third day of irrigation from fully matured young leaves (facing the sun) and averages were calculated at 10–12 a.m. (local time).

- Growth and chlorophyll contents: After the treatments, plant height at maturity and leaf area index was recorded by using a meter rod and the portable laser leaf area meter model CI-2002L (CID BioScience, USA), respectively. For computing the chlorophyll index, the chlorophyll meter model CL-01 (Hansatech Instruments Ltd., UK) was used (Raza et al. 2017).
- Water relations in cotton: Fully expanded youngest leaf was selected to calculate the fresh weight (FW) of the cotton leaf. To compute the turgid weight (TW) of the cotton leaf, it was soaked for 18–20 h at 25°C and was dried by using tissue paper. The leaf dry weight (DW) was measured by keeping the leaf in the oven for 3 days at 70°C and the leaf relative water content (LRWC) was calculated using the following calculation :
  $$ \mathrm{LRWC}\ \left(\%\right)=\left(\ \mathrm{FW}-\mathrm{DW}\right)/\left(\mathrm{TW}-\mathrm{DW}\right)\times 100 $$

Excised leaf water loss (*ELWL*) from cotton leaf was also calculated using the following calculation:

$$ \mathrm{ELWL}\ \left(\%\right)=\left(\ \mathrm{FW}-\mathrm{WW}\right)/\left(\ \mathrm{DW}\times WW\right) $$

where *WW* is the wilted weight.

Water potential apparatus (Chas W. Cook Div., England) was used to measure the water potential (-MPa) of the cotton leaf. A fully stretched leaf at the top was selected and a vapor pressure osmometer (Wescor 5520, Logan, USA) was used to determine the osmotic potential (-MPa) of the leaf from the frozen sap of crushed leaf. Turgor pressure (MPa) of the leaf was computed by subtracting the water potential (ψw) from osmotic potential (ψs):TP = *OP* − *WP* (Raza et al. 2017).

(iii) Leaf gas exchange: The portable photosynthesis apparatus (Li-COR-LI 6250) was used for the measurement of leaf stomatal conductance and photosynthetic rate (Raza et al. 2017).
(iv) Leaf abscisic acid (ABA): The leaf ABA was determined by using the protocols described by Speirs et al. (2013).
(v) Total sugars and proline: The soluble sugars and proline contents in the cotton leaves were measured by adopting the procedures of Nelson (1944) and Bates et al*.* (1973), respectively.
(vi) Soil moisture (%): Top soil (0–20 cm) moisture content was determined gravimetrically after oven drying (105 ^0^C), the samples to constant weight (Iqbal et al. 2020) before the application of irrigation water.
(vii) Antioxidant enzyme activities: To determine the antioxidants activity, first, the protein content was measured in cotton leaves by taking bovine serum album as standard (Bradford, 1976), and afterward the measurements of antioxidants were made accordingly. The ascorbate peroxidase (APX), catalase (CAT), peroxidase (POD), and superoxide dismutase (SOD) activities were determined according to Anderson et al. (1992), Beers and Sizer (1952), Maehly and Chance (1954), and Giannopolitis and Ries (1977), respectively.
(viii) Quality traits and water use efficiency: The cotton quality parameters (e.g., fiber length, strength, and fineness) were recorded on the HVI instrument, and the percentage of “ginning out-turn” was calculated using the following calculation:
  $$ \mathrm{Ginning}\ \mathrm{out}-\mathrm{turn}\ \left(\%\right)=\left(\mathrm{Weight}\ \mathrm{of}\ \mathrm{lint}\ \left(\mathrm{g}\right)/\mathrm{Weight}\ \mathrm{of}\ \mathrm{seed}\ \mathrm{cotton}\ \left(\mathrm{g}\right)\right)\times 100 $$

Besides, the water use efficiency (kg ha^-1^ mm^-1^) of cotton was calculated using the following calculation:

$$ \mathrm{Water}\ \mathrm{use}\ \mathrm{efficiency}=\mathrm{Seed}\ \mathrm{cotton}\ \left(\mathrm{kg}\ \mathrm{ha}-1\right)/\mathrm{Total}\ \mathrm{water}\ \mathrm{application}\ \left(\mathrm{mm}\right) $$

### Statistical analysis

Data were analyzed statistically by using STATISTICS software (version 9.2, Analytical Software, Saint Paul, MN) and means were compared by the least significant difference (LSD) at a 5% probability level (Steel et al. 1997). The principal component analysis (PCA) was performed with Origin Pro 9.1 software (Origin-Lab Corporation, Northampton, MA) to describe patterns of variations among control and treated plants. Results of this analysis were examined with a biplot graph developed from principal components (PC 1) and (PC 2) derived from PCA. This biplot graph integrated the different scales of study to demonstrate the potential of photosynthesis, antioxidants, osmolytes, and water-related traits to explain the cotton yield variations along with respect to PRD and mulches regimes. MetaboAnalyst (version 4.0) was used for developing a heat map with hierarchical clustering.

## Results

### Growth, yield, weeds, and fiber quality-related parameters

The effect of different mulches and irrigation intervals on plant height and yield-related parameters of cotton is presented in Table 4. The maximum plant height (144cm) was observed for the year 2017 under wheat straw mulch (WSM). Maximum plant height (160cm) was observed in FI and minimum in PRD (104cm). Considering the interactive effect, the maximum plant height (172cm) was observed in WSM+FI and the lowest in NM+PRD (93 cm). The same trend for plant height was also observed in 2018. Considering the data related to leaf area index (LAI) in 2017, using WSM showed the highest LAI (2.30) followed by BPM (2.10), and the minimum LAI was observed in CSM (1.81). Considering irrigation regimes, the maximum LAI (2.37) was recorded in FI treatment and the minimum (1.48) was in PRD. Based on our results, there was no statistically significant difference among the interactions for the year 2017 but present in 2018.

**Table 4:** Effect of different mulching and irrigation treatments on growth, yield, and weed-related parameters of cotton. Plant height (PH, cm), Leaf area index (LAI), number of bolls per plant (NB), number of sympodial branches per plant (NSB),100-bolls weight (HBW, g), Seed cotton yield (SCY, kg ha^-1^), biological yield (BY, kg ha^-1^), harvest index (HI, %), lint yield (LY, kg ha^-1^), number of weeds per meter square (NW), and weed biomass (WB, g)

Year	Treatments		PH	LAI	NB	NSB	HBW	SCY	BY	HI	LY	NW	WB
2017	FI	NM	145.3^D^	2.05^B^	38.00^D^	20.00^CD^	328.0^D^	3342.3^D^	8521^CD^	39.22^AB^	1652.2^D^	135.33^A^	67.00^A^
		BPM	165.1^B^	2.60^A^	45.00^B^	23.67^B^	365.0^B^	4143.0^B^	9931^AB^	41.72^AB^	1492.7^B^	21.33^G^	12.93^EF^
		WSM	172.3^A^	2.74^A^	47.33^A^	27.33^A^	372.3^A^	4456.7^A^	10529^A^	42.33^AB^	1652.2^A^	35.67^E^	24.24^D^
		CSM	158.3^C^	2.10^B^	41.00^C^	22.00^BC^	355.7^C^	3842.7^C^	9145^BC^	42.01^AB^	1314.7^C^	54.00^C^	37.36^C^
	PRD	NM	93.3^H^	1.01^D^	27.00^H^	11.00^G^	231.3^H^	1934.0^H^	4522^F^	42.77^AB^	565.7^H^	83.00^B^	49.67^B^
		BPM	108.4^F^	1.51^C^	33.33^F^	15.00^EF^	283.0^F^	2761.3^F^	6279^E^	45.92^A^	856.7^F^	12.00^H^	9.03^F^
		WSM	115.4^E^	1.87^BC^	35.33^E^	17.33^DE^	292.3^E^	2958.7^E^	7941^D^	37.26^B^	930.8^E^	26.00^F^	17.03^E^
		CSM	100.7^G^	1.53^C^	29.33^G^	12.67^FG^	277.0^G^	2520.0^G^	6849^E^	36.79^B^	774.4^G^	40.67^D^	26.86^D^
LSD (*P* ≤ 0.05)			1.56	0.40	2.06	3.56	2.54	32.86	998.4	8.42	45.00	3.72	4.02
2018	FI	NM	140.6^D^	2.22^C^	36.00^D^	18.00^C^	325.0^D^	3229.0^D^	8427^D^	38.32^ABC^	985.0^D^	140.0^A^	67.26^A^
		BPM	160.0^B^	2.55^B^	43.00^B^	23.00^B^	360.0^B^	4047.0^B^	9749^B^	41.51^AB^	1349.0^B^	22.7^G^	14.46^G^
		WSM	169.8^A^	2.75^A^	45.00^A^	25.67^A^	368.0^A^	4248.3^A^	10237^A^	41.50^AB^	1533.0^A^	38.0^E^	26.40^E^
		CSM	153.7^C^	2.49^B^	40.00^C^	21.00^B^	355.0^B^	3731.0^C^	8985^C^	41.52^AB^	1286.7^C^	56.67^C^	37.20^C^
	PRD	NM	95.1^H^	1.10^G^	25.00^H^	9.33^F^	255.0^G^	1896.7^H^	4437^H^	42.74^A^	502.0^G^	88.0^B^	54.00^B^
		BPM	118.2^E^	1.74^E^	31.00^F^	15.00^D^	279.0^E^	2616.7^F^	6971^F^	37.53^BCD^	764.7^F^	15.0^H^	10.01^H^
		WSM	110.2^F^	1.84^D^	34.00^E^	17.00^CD^	290.0^D^	2863.7^E^	7738^E^	32.72^D^	920.7^E^	30.0^F^	18.00^F^
		CSM	104.3^G^	1.50^F^	27.00^G^	12.00^E^	271.0^F^	2444.0^G^	6748^G^	36.22^CD^	742.3^F^	41.0^D^	30.16^D^
LSD (*P* ≤ 0.05)			1.98	0.08	1.48	2.27	7.61	90.43	61.15	4.64	60.54	3.02	1.09
Significance													
2017	Irrigation		**	*	**	**	**	**	**	ns	**	**	**
	Mulch		**	**	**	**	**	**	**	ns	**	**	**
	Mulch × irrigation		*	ns	ns	ns	**	ns	ns	ns	**	**	**
2018	Irrigation		**	**	**	**	**	**	**	**	**	**	**
	Mulch		**	**	**	**	**	**	**	ns	**	**	**
	Mulch × irrigation		**	**	ns	ns	**	ns	**	**	*	**	**

Significant differences are indicated by an asterisk (^∗^); ^∗^*P* ≤ 0.05, ^∗∗^*P* ≤ 0.01; *NS*, non-significant. *FI* full irrigation, *PRD* partial root-zone drying, *NM* no mulch, *BPM* black plastic mulch, *WSM* wheat straw mulch (WSM), *CSM* cotton sticks mulch. Superscripted capital letters show significance among different mulches and irrigation treatments

Both the factors (mulches and irrigations) had a significant effect on the number of bolls per plant of cotton (Table 4). WSM achieved a higher number of bolls per plant followed by BPM (39) and a minimum number of bolls per plant was counted in CSM (35). For irrigation treatments, a higher number of bolls per plant was recorded in FI, while minimum (31) was attained in PRD. Among the interactions, there was no statistically significant difference for both years and the resulting trend was similar.

Both factors mulches and irrigation intervals had a significant effect on the number of sympodial branches per plant of cotton (Table 4). Among the mulches, WSM attained the more number of sympodial branches per plant (22) followed by BPM (19) and the lowest number of sympodial branches per plant was recorded in CSM (17). Intended for irrigation intervals, more number of sympodial branches per plant (23) was counted in FI and less number (14) was recorded in PRD and the same trend was observed for the year 2018. Among the interactions, there was no statistically significant difference for both growing seasons.

Data regarding 100-bolls weight is shown in (Table 4) shows that both treatments had a significant effect on 100-bolls weight (g) of cotton. WSM attained the more 100-bolls weight (332 g) followed by BPM (324 g) and the minimum 100-bolls weight was recorded in CSM (316 g). For irrigation (PRD vs. FI) intervals, more 100-bolls weight (355 g) was counted in FI and minimum (270 g) was in PRD. Among the interactions, there was a statistically significant difference. Maximum 100-bolls weight (372g) was counted in WSM+FI and minimum was in NM+FI (231 g). The same trend was also found in 2018.

Both factors (mulches and irrigation) had a statistically significant effect on seed cotton yield (kg/ha) (Table 4). Among the mulch treatments, WSM attained the higher value of seed cotton yield (3707 kg/ha) followed by BPM (3452 kg/ha) and minimum seed cotton yield was recorded in CSM (3181 kg/ha). For irrigation intervals, more value of seed cotton yield (3946 kg/ha) was measured in FI and less value (2543 kg/ha) was measured in PRD. Among the interactions, there was a statistically significant difference in 2017. Maximum seed cotton yield (4456.7 kg/ha) was calculated in WSM+FI and minimum was computed in NM+PRD (1934.0 kg/ha). Interactions of treatments were found non-significant in 2018.

Both factors have a significant effect on biological yield (kg/ha) (Table 4). Among the mulches, WSM attained the maximum biological yield (9234 kg/ha) followed by BPM (8104.8 kg/ha) and the minimum biological yield was recorded in CSM (7997 kg/ha). For irrigation intervals, maximum biological yield (9531 kg/ha) was recorded in FI and minimum (6397 kg/ha) was recorded in PRD. Interactions for biological yield had no significant effect for both the factors in the year 2017 but were found statistically significant in 2018. Data regarding harvest index is shown in Table 4; both factors had no significant effect on the harvest index (%). For irrigation intervals, harvest index was statistically non-significant in both irrigation levels (FI and PRD) for the first year (2017) but statistically significant for the second year (2018). Interactions of both treatments were non-significant for 2017 and statistically significant for 2018. Table 4 indicates the significant effect of both factors on the lint yield (kg/ha) of cotton. Among the mulch treatments, WSM got the more value of lint yield (1291 kg/ha) followed by BPM (1174 kg/ha) and minimum lint yield was recorded in CSM (1044 kg/ha). For irrigation intervals more lint yield (1382 kg/ha) was recorded in FI and less (781 kg/ha) was counted in PRD. The tendency of results for lint yield was similar in 2018. Among the interactions, there was also a statistically significant difference. Maximum lint yield (1652 kg/ha) was recorded in WSM+FI and minimum was recorded in NM+PRD (565 kg/ha).

Table 4 represents the data related to weeds infestation (number of weeds/m^2^). Among the mulches, more infestation (109.17) was recorded in NM (no mulch) followed by CSM (47.33), and the minimum infestation (16.67) was recorded in BPM. For irrigation levels, a maximum number of weeds/m^2^ (61.58) were recorded in FI and minimum weeds (40.41) were observed in PRD. Among the interaction maximum (135.33) number of weeds were achieved in NM+FI and a minimum (12.00) in BPM+PRD. Data related to weed biomass is shown in Table 4 indicate that both factors had a significant effect on weed biomass (g/m^2^). Among the mulches, more weed biomass (58.33 g/m^2^) was recorded in NM followed by CSM (32.11g/m^2^) and less weed biomass (10.9811g/m^2^) was recorded in BPM. For irrigation levels, more weed biomass (35.38 g/m^2^) was recorded in FI and less weed biomass (25.65 g/m^2^) was observed in PRD for both years. Among the interaction, a higher value (67.0011g/m^2^) of weed biomass was achieved in NM+FI and minimum (9.0311g/m^2^) in BPM+PRD. Weed numbers and biomass results’ trend for the second year was in accordance with first-year results for all the treatments.

Ginning out turn (GOT) of cotton for both treatments is shown in Table 5 indicating that both the factors (mulches and irrigations) had a significant effect on GOT. Among the mulches, WSM achieved the higher value of GOT (34.26 %) followed by BPM (33.52 %) and minimum GOT was recorded in CSM (32.97 %). For irrigation intervals, more GOT (35.09 %) was counted in FI and less (30.61 %) was in PRD. Among the interactions, there was also a statistically significant difference. Maximum GOT (37.07 %) was measured in WSM+FI and the lowest value was counted in NM+PRD (29.25 %) but it was non-significant in 2018. The fiber length of cotton (Table 5) is also significant for both factors. Among the mulch treatments, WSM got the maximum fiber length (27.33 mm) followed by BPM (27.05 mm), and the minimum fiber length was recorded in CSM (26.82 mm). For irrigation intervals, higher fiber length (27.76 mm) was observed in FI and less (25.93 mm) in PRD. Among the interactions, there was a statistically non-significant difference during 2017 but it was highly significant in 2018.

**Table 5:** Effect of different mulching and irrigation treatments on quality, water-related, and soil moisture of cotton. Ginning out turn (GOT, %), fiber length (FL, mm), fiber strength (FS,g tex^-1^), fiber fineness (FF, micronaire), excised leaf water loss (ELWL, %), leaf relative water contents (LRWC, %), leaf water potential (LWP, -MPa), leaf osmotic potential (LOP, -MPa), leaf turgor potential (LTP, MPa), and soil moisture (SM, %)

Year	Treatments		GOT	FL	FS	FF	ELWL	LRWC	LWP	LOP	LTP	SM
2017	FI	NM	32.05^D^	27.04^C^	30.65^D^	2.89^C^	1.73^BC^	80.50^D^	1.72^G^	11.50^H^	9.78^H^	15.50^D^
		BPM	36.03^B^	27.95^B^	31.45^B^	3.91^AB^	2.24^A^	86.50^B^	1.77^F^	14.03^F^	12.23^E^	23.50^A^
		WSM	37.07^A^	28.32^A^	31.95^A^	4.22^A^	2.17^AB^	89.50^A^	1.83^E^	15.50^D^	13.70^D^	19.70^B^
		CSM	35.21^C^	27.75^B^	31.02^C^	3.20^BC^	1.43^CD^	82.50^C^	1.42^H^	12.50^G^	10.66^G^	17.60^C^
	PRD	NM	29.25^H^	25.32^F^	29.03^H^	4.52^A^	0.94^E^	66.50^H^	2.80^B^	15.03^E^	12.17^F^	6.06^H^
		BPM	31.02^F^	26.15^DE^	30.02^F^	4.65^A^	1.38^CDE^	73.50^F^	2.49^C^	18.03^B^	15.58^B^	10.90^E^
		WSM	31.45^E^	26.35^D^	30.35^E^	4.07^A^	1.50^CD^	77.50^E^	2.97^A^	20.50^A^	17.52^A^	9.83^F^
		CSM	30.73^G^	25.90^E^	29.91^G^	4.51^A^	1.15^DE^	70.16^G^	2.22^D^	16.50^C^	14.30^C^	8.00^G^
LSD (*P* ≤ 0.05)			0.10	0.25	0.05	0.78	0.46	1.01	0.02	0.02	0.02	0.68
2018	FI	NM	30.50^D^	26.04^D^	29.63^D^	2.75^H^	1.65^D^	76.33^D^	1.64^G^	9.50^E^	7.86^F^	14.50^D^
		BPM	33.32^BC^	26.96^B^	30.45^B^	3.25^F^	1.94^B^	81.13^B^	1.72^F^	12.03^D^	10.31^D^	22.50^A^
		WSM	36.08^A^	27.35^A^	30.98^A^	3.07^G^	2.03^A^	85.16^A^	1.76^E^	13.50^C^	11.73^C^	18.30^B^
		CSM	34.48^B^	26.65^C^	30.01^C^	3.90^E^	1.86^C^	78.50^C^	1.37^H^	10.50^E^	9.13^E^	16.20^C^
	PRD	NM	26.45^E^	24.14^H^	28.02^H^	4.20^D^	0.98^H^	62.53^G^	2.75^B^	12.00^B^	9.24^DE^	8.00^F^
		BPM	29.21^D^	25.20^F^	29.03^F^	4.46^C^	1.25^F^	69.36^F^	2.36^C^	16.00^B^	13.62^B^	10.67^E^
		WSM	32.13^C^	25.40^E^	29.40^E^	4.64^A^	1.34^E^	72.23^E^	2.91^A^	18.44^A^	15.53^A^	10.30^E^
		CSM	30.36^D^	24.85^G^	28.90^G^	4.52^B^	1.15^G^	64.50^G^	2.15^D^	14.47^C^	12.32^C^	8.30^F^
LSD (*P* ≤ 0.05)			1.47	0.07	0.07	0.03	0.02	1.47	0.03	0.52	0.52	0.85
2017	Irrigation		**	**	**	**	*	**	**	**	**	**
	Mulch		**	**	**	ns	**	**	**	**	**	**
	Mulch × irrigation		**	ns	**	*	ns	*	**	**	**	**
2018	Irrigation		**	**	**	**	**	**	**	**	**	**
	Mulch		**	**	**	**	**	**	**	**	**	**
	Mulch × irrigation		ns	*	**	**	ns	ns	**	**	**	**

Significant differences are indicated by an asterisk (^∗^); ^∗^*P* ≤ 0.05, ^∗∗^*P* ≤ 0.01; *NS*, non-significant. *FI* full irrigation, *PRD* partial root-zone drying, *NM* no mulch, *BPM* black plastic mulch, *WSM* wheat straw mulch, *CSM* cotton sticks mulch. Superscripted capital letters show significance among different mulches and irrigation treatments

Among the mulches, WSM got the more strength of fiber (31.15 g tex^-1^) followed by BPM (30.73 g tex-^1^) and minimum fiber strength was recorded in CSM (30.46 g tex^-1^). For irrigation intervals, more value of fiber strength (31.26 g tex^-1^) was recorded in FI and less value (29.83 g tex^-1^) was recorded in PRD. For interactions, there was a statistically significant difference. Maximum fiber strength (31.95 g tex^-1^) was recorded in WSM+FI and minimum was recorded in NM+PRD (29.03 g tex^-1^). Treatments had a significant effect on the fiber fineness of cotton (Table 5). BPM attained the maximum fiber fineness (4.28 micronaire) which was at par with WSM (4.15 micronaire) and CSM (3.85 micronaire) but the minimum value of fiber fineness was in NM (3.71 micronaire). For irrigation intervals, maximum fiber fineness (4.44 micronaire) was achieved in PRD and the minimum value (3.55 micronaire) in FI (Table 5). Among the interactions, there was a statistically significant difference. Maximum fiber fineness (4.65 micronaire) was recorded in BPM+PRD and minimum was recorded in NM+FI (2.89 micronaire). The tendency of results for fiber fineness was also the same during the second year (2018).

### Water-related parameters and soil moisture content measurements

Considering data related to the excised leaf water loss for both treatments of irrigation and mulch applications for the year of 2017–2018 presented in Table 5 that shows significant effects of each one separately. Among the treatments of mulches, WSM shows more value of excised leaf water loss (1.83%) in comparison to BPM (1.81%), and the lowest value was recorded using the CSM (1.29%). Considering irrigation techniques, the maximum value (1.89%) was recorded for FI and the minimum (1.24%) for PRD irrigation technique. The year 2018 shows the same trend as 2017. Leaf relative water content is also significantly affected by both of the treatments and the resulting trend was the same for both years of the experiment. For various mulches, WSM shows more value (83.50%) compared to other mulch applications. Irrigation treatments had also significant results and more value (84.75%) was recorded from FI and the minimum value (71.91%) was recorded for PRD. Integration of both treatments was also found statistically significant, where the highest value (89.50%) was measured from WSM+FI and the lowest value was observed from NM+PRD (66.50%). Considering the leaf water potential (LWP) in 2017, WSM among the mulch applications showed the maximum negative value (−2.40 MPa) followed by NM treatment (−2.26 MPa), and the minimum LWP was observed in CSM (−1.82 MPa). For irrigation treatments, the maximum negative value (−2.60 MPa) was measured via PRD and the minimum value (−1.68MPa) was recorded via FI. Integration of both of the treatments was also found significant. Among the interaction of the treatments, the more negative value (−2.97 MPa) was recorded for WSM+PRD. Similar results were also found in the next growing season (2018).

Both factors (mulches and irrigation) had a statistically significant effect on leaf osmotic potential (LOP) in cotton (Table 5). Mulch treatment, WSM achieved the higher negative value of leaf osmotic potential (−18.00 MPa) in comparison to BPM (-16.03 MPa) and CSM (−14.50 MPa). For irrigation treatments, higher negative value of LOP (−17.51 MPa) was recorded for PRD and a lower (−13.38 MPa) value of LOP was recorded for FI. Considering the leaf turgor potential (LTP) in 2017 in Table 4, among the mulch treatments, WSM showed a higher value of LTP (15.61MPa) in comparison to BPM (13.90MPa) and the lowest LTP value was observed for CSM (12.48 MPa). A higher LTP value for irrigation (14.89 MPa) levels was seen in PRD and less LTP value (11.59 MPa) was attained in FI. Among the interactions between the treatments, the highest LTP (17.52 MPa) was recorded for WSM+PRD and the lowest was from NM+FI (9.78MPa). The resulting trend for osmotic and turgor potential was the same in the year 2018.

Considering the soil moisture data for 2017–2018 in Table 5, both of the factors had a significant effect. Among the mulch treatments, BPM showed the maximum soil moisture (17.20%) followed by WSM (14.76%), and the minimum soil moisture was recorded in CSM (12.80%). Results of irrigation intervals showed more soil moisture (19.07%) for FI and the lowest (8.69%) were recorded from PRD. Interaction of both of the treatments also showed significant results on soil moisture. Among these interactions, the highest value (23.50%) was measured for BPM+FI and the lowest value was recorded with NM+PRD (6.06%). The tendency of results for the year 2018 was similar to 2017 for soil moisture percentage.

### Water use efficiency and physio-biochemical traits of the cotton crop

Both studied factors had a significant effect on water use efficiency (kg ha^-1^ mm^-1^) of the cotton crop (Table 6). Among the mulch treatments, WSM got the more water use efficiency value (4.93 kg ha^-1^ mm^-1^) followed by BPM (4.59 kg ha^-1^ mm^-1^) and less value of water use efficiency was recorded in CSM (4.22 kg ha^-1^ mm^-1^). For irrigation intervals, less value of water use efficiency (4.84 kg ha^-1^ mm^-1^) was recorded in PRD and minimum (3.75 kg ha^-1^mm^-1^) was recorded in FI. The tendency of a similar result was also recorded in 2018. Among the interaction, there was a statistically significant difference in 2017. Maximum water use efficiency (5.63 kg ha^-1^ mm^-1^) was counted in WSM+PRD and minimum was recorded in NM+FI (3.68 kg ha^-1^ mm^-1^). Interactions for 2018 were found non-significant.

**Table 6:** Effect of different mulching and irrigation treatments on water use efficiency, osmolytes, and antioxidants related parameters of cotton. Water use efficiency (kg ha^-1^ mm^-1^), leaf chlorophyll contents (LCC, %), stomatal conductance (SC, mmolm^-2^s^-1^), photosynthetic rate (Pn, μmolm^-2^s^-1^), total sugar contents (TSC, mg g^-1^), leaf abscisic acid (ABA, ng g^-1^), proline content (μmol g^-1^), superoxide dismutase (SOD, units mg^-1^ protein), peroxidase (POD, units mg^-1^ protein), catalase (CAT, units mg^-1^ protein), and ascorbate peroxidase (APX, units mg^-1^ protein)

Year	Treatments		WUE	LCC	SC	Pn	TSC	ABA	Proline	SOD	POD	CAT	APX
2017	FI	NM	3.13^G^	41.00^D^	388.0^B^	16.00^D^	13.50^H^	168.3^D^	3.03^H^	1.20^H^	12.80^H^	155.0^H^	1.20^G^
		BPM	3.94^E^	47.50^B^	377.0^C^	20.00^B^	14.03^F^	189.7^CD^	3.70^F^	1.60^F^	18.46^F^	181.0^F^	1.60^E^
		WSM	4.24^D^	49.33^A^	395.0^A^	23.00^A^	15.50^E^	204.3^C^	4.10^E^	1.90^E^	21.50^E^	195.7^E^	1.90^D^
		CSM	3.65^F^	44.00^C^	355.0^D^	17.83^C^	13.90^G^	203.0^C^	3.30^G^	1.40^G^	16.10^G^	165.0^G^	1.40^F^
	PRD	NM	3.68^F^	30.16^H^	302.0^F^	11.80^F^	18.03^D^	902.3^A^	6.70^D^	2.30^D^	26.49^D^	215.7^D^	2.20^C^
		BPM	5.25^B^	37.00^F^	280.0^G^	13.30^EF^	21.03^B^	883.7^AB^	7.80^B^	3.10^B^	36.60^B^	274.7^B^	2.53^B^
		WSM	5.63^A^	39.00^E^	310.0^E^	14.33^E^	23.50^A^	861.7^B^	8.83^A^	3.60^A^	43.40^A^	305.3^A^	2.90^A^
		CSM	4.79^C^	33.90^G^	260.0^B^	12.21^F^	19.90^C^	877.7^AB^	7.30^C^	2.80^C^	33.16^C^	245.0^C^	2.30^C^
LSD (P ≤ 0.05)			0.04	1.92	2.82	1.02	0.07	37.17	0.18	0.19	1.17	4.65	0.13
2018	FI	NM	3.07^D^	38.33^D^	382.0^B^	15.50^D^	14.90^G^	198.0^F^	4.06^G^	1.30^G^	12.10^H^	160.0^H^	1.50^D^
		BPM	3.85^C^	44.06^B^	370.6^C^	19.43^B^	16.00^F^	215.0^E^	4.70^F^	1.70^E^	18.20^F^	185.0^F^	1.60^D^
		WSM	4.04^BC^	47.56^A^	388.3^A^	22.50^A^	17.50^E^	204.0^F^	5.16^E^	1.10^H^	20.50^E^	200.0^E^	2.03^C^
		CSM	3.54^CD^	40.36^C^	348.0^B^	17.20^C^	15.10^G^	212.6^E^	4.50^F^	1.50^F^	16.50^G^	170.0^G^	1.70^D^
	PRD	NM	3.61^CD^	27.60^G^	250.0^G^	11.20^F^	20.50^D^	970.0^A^	7.70^D^	2.50^C^	28.00^D^	220.0^D^	2.50^B^
		BPM	4.97^A^	34.53^E^	296.0^F^	12.16^EF^	23.00^B^	885.0^D^	8.80^B^	3.30^B^	39.00^B^	280.0^B^	2.90^A^
		WSM	4.81^A^	37.53^D^	303.7^E^	13.05^E^	24.90^A^	915.0^C^	9.80^A^	3.80^A^	45.50^A^	310.0^A^	3.04^A^
		CSM	4.65^AB^	30.30^F^	251.7^G^	11.92^EF^	21.50^C^	935.0^B^	8.20^C^	2.10^D^	35.53^C^	250.0^C^	2.76^AB^
LSD (P ≤ 0.05)			0.69	1.35	3.45	1.81	0.79	7.14	0.27	0.09	1.02	1.63	0.30
Significance													
2017	Irrigation		**	**	**	**	**	**	**	**	**	**	**
	Mulch		**	**	**	**	**	ns	**	**	**	**	**
	Mulch × irrigation		**	ns	**	**	**	*	**	**	**	**	ns
2018	Irrigation		**	**	**	**	**	**	**	**	**	**	**
	Mulch		**	**	**	**	**	**	**	**	**	**	*
	Mulch × irrigation		ns	ns	**	**	*	**	**	**	**	**	ns

Significant differences are indicated by asterisk (^∗^); ^∗^*P* ≤ 0.05, ^∗∗^*P* ≤ 0.01; *NS*, non-significant. *FI* full irrigation, *PRD* partial root-zone drying, *NM* no mulch, *BPM* black plastic mulch, *WSM* wheat straw mulch, *CSM* cotton sticks mulch. Superscripted capital letters show significances among different mulches and irrigation treatments

Considering the leaf chlorophyll contents, among the mulch treatments, WSM resulted in the maximum value of leaf chlorophyll content (44.16%) followed by BPM (42.25%), and the lowest value was observed for CSM (38.95%). Considering both of the irrigation regimes, a maximum value (45.45%) was recorded for FI and the lowest (35.01%) was recorded for PRD (Table 6). Similar results were also found in 2018 and in general the interactions for chlorophyll content were not statistically significant for both of the years.

Table 6 shows that both treatments of mulch application and irrigation techniques had a statistically significant effect on stomatal conductance in cotton leaves for 2017–2018. For various mulches used in this research, WSM showed more values of stomatal conductance (352.50 mmol m^-2^s^-1^) followed by BPM (328.50 mmol m^-2^s^-1^), and the minimum value was recorded from CSM (307.50 mmol m^-2^s^-1^). Irrigation intervals resulted in maximum stomatal conductance (378.75 mmolm^-2^s^-1^) for FI and minimum (288.00 mmolm^-2^s^-1^) for PRD technique. Considering the integration of the treatments, the maximum stomatal conductance (395.00 mmol m^-2^s^-1^) was measured from WSM+FI and the minimum value was attained from CSM+PRD (260.00 mmolm^-2^s^-1^). The same result trend was also recorded for 2018. Considering the photosynthetic rate (Table 6), both factors had a significant effect. Among the mulch treatments, WSM showed the highest value of photosynthetic rate (18.67 μmolm^-2^s^-1^) followed by BPM (16.65 μmolm^-2^s^-1^), and the lowest value of photosynthetic rate was recorded in CSM (15.02 μmolm^-2^s^-1^). For irrigation intervals, a higher value of photosynthetic rate (19.20 μmol m^-2^s^-1^) was observed in FI treatment and a lower value (12.91 μmol m^-2^s^-1^) was measured in PRD. Interactions between the two factors had also a statistically significant effect where the maximum photosynthetic rate (23.00 μmol m^-2^s^-1^) was observed from WSM+FI and the lowest value was attained from NM+PRD (11.80 μmol m^-2^s^-1^). Findings for both of the years of the experiments were similar in tendency.

Both factors of mulch applications and irrigation techniques had a statistically significant effect on total sugar contents in cotton leaves (Table 6). Considering the mulch applications, the maximum value of total sugars (19.50 mg g^-1^) was recorded for WSM in comparison to BPM (17.53 mg g^-1^), and the lowest value (15.76 mg g^-1^) was analyzed for CSM. Considering the irrigation effect, the maximum total sugars (20.61 mg g^-1^) resulted from PRD and the minimum value (14.23 mg g^-1^) from FI. The interaction effect of mulch application and irrigation techniques was also significant and more value (23.50 mg g^-1^) was achieved from WSM+PRD and the lowest (13.50 mg g^-1^) was attained from NM+FI. A similar trend of results was also found from the second year. Data on leaf abscisic acid (ABA) concentration (ng g^-1^) in cotton leaves (Table 6) showed that the different mulches have no significant effect on the ABA concentrations in cotton leaves but both irrigation techniques had statistically highly significant effect. The maximum (881.33 ng g^-1^) value of ABA was found under PRD and the minimum (191.33 ng g^-1^) was recorded from the FI. Integration of both of the treatments had a significant effect where the more (902.33 ng g^-1^) value of ABA was observed under BPM+PRD and the lowest (168.33 ng g^-1^) was attained from BPM+FI. Table 6 shows data on proline contents in cotton leaves considering various mulches at two irrigation techniques. Among the mulches, the highest value of proline content (6.46 μmol g^-1^) was recorded from WSM followed by BPM (5.75 μmol g^-1^) and the minimum (4.86 μmol g^-1^) was seen from CSM. Considering irrigation techniques, the highest proline content value (7.65 μmolg^-1^) was from PRD and the minimum (3.53 μmol g^-1^) was recorded from the FI treatment. Considering the integration of the treatments, the maximum (8.83 μmol g^-1^) value was achieved from WSM+PRD and the minimum (3.03 μmol g^-1^) in NM+FI. Abscisic acid and proline have a similar tendency of results in 2018 (Table 6).

Table 6 shows the data concerning superoxide dismutase (SOD) in cotton using various mulches and irrigation treatments for both experiment years. Among the mulches, higher SOD activity (2.75 units/mg protein) was measured in WSM as compared to BPM (2.35 units/mg protein), and the lowest SOD activity value (1.75 units/mg protein) was in CSM. Considering the irrigation treatments, the highest value (2.95 units/mg protein) resulted from PRD, and the minimum one (1.52 units/mg protein) was recorded from FI. Interactions between mulches and irrigation techniques were also significant. Among the interactions, the maximum value was from WSM+PRD (3.6 units/mg protein) and the minimum was from NM+FI (1.2 units/mg protein). Peroxidase (POD) values in cotton leaves are shown in Table 6. The WSM got the more activity of POD (32.45 units/mg protein) in comparison to BPM (27.53 units/mg protein) and the lowest value (19.64 units/mg protein) was measured for CSM. The PRD got the most value of POD (34.91 units/mg protein) and FI treatment got the lowest activity of POD (17.21 units/mg protein) for irrigation. Among the interactions, higher activity of POD was analyzed in WSM+PRD (43.40 units/mg protein) and lower value of POD in NM+FI (12.80 units/mg protein).

Data about catalase (CAT) in cotton leaves using various mulches and irrigation levels also showed a significant difference. Among the mulches, higher activity of CAT (250.50 units/mg protein) was analyzed in WSM than BPM (227.83 units/mg protein) and the lowest activity of CAT (185.33 units/mg protein) was in CSM. Intended for irrigation levels, more activity of CAT (260.17 units/mg protein) was in PRD and less activity value of CAT (174.17 units/mg protein) was analyzed in FI. Among the interactions, more activity value of CAT was observed in WSM+PRD (305.33 units/mg protein) and less in NM+FI (155.00 units/mg protein). Ascorbate peroxidase (APX) values in cotton leaves are shown in Table 6. Both the factors had a significant effect on APX. WSM got the maximum activity of APX (2.40 units/mg protein) than BPM (2.06 units/mg protein) and the lowest activity of APX (1.70 units/mg protein) was analyzed in CSM. The PRD got the highest activity value of APX (2.48 units/mg protein) and FI got the lowest activity value of APX (1.52 units/mg protein) for irrigation. Interactions were statistically non-significant for both factors. SOD, POD, CAT, and APX have the same results in the second year of research as got in the first year.

### Data mining using heat map and principal component analysis (PCA) techniques

**Fig. 2:**
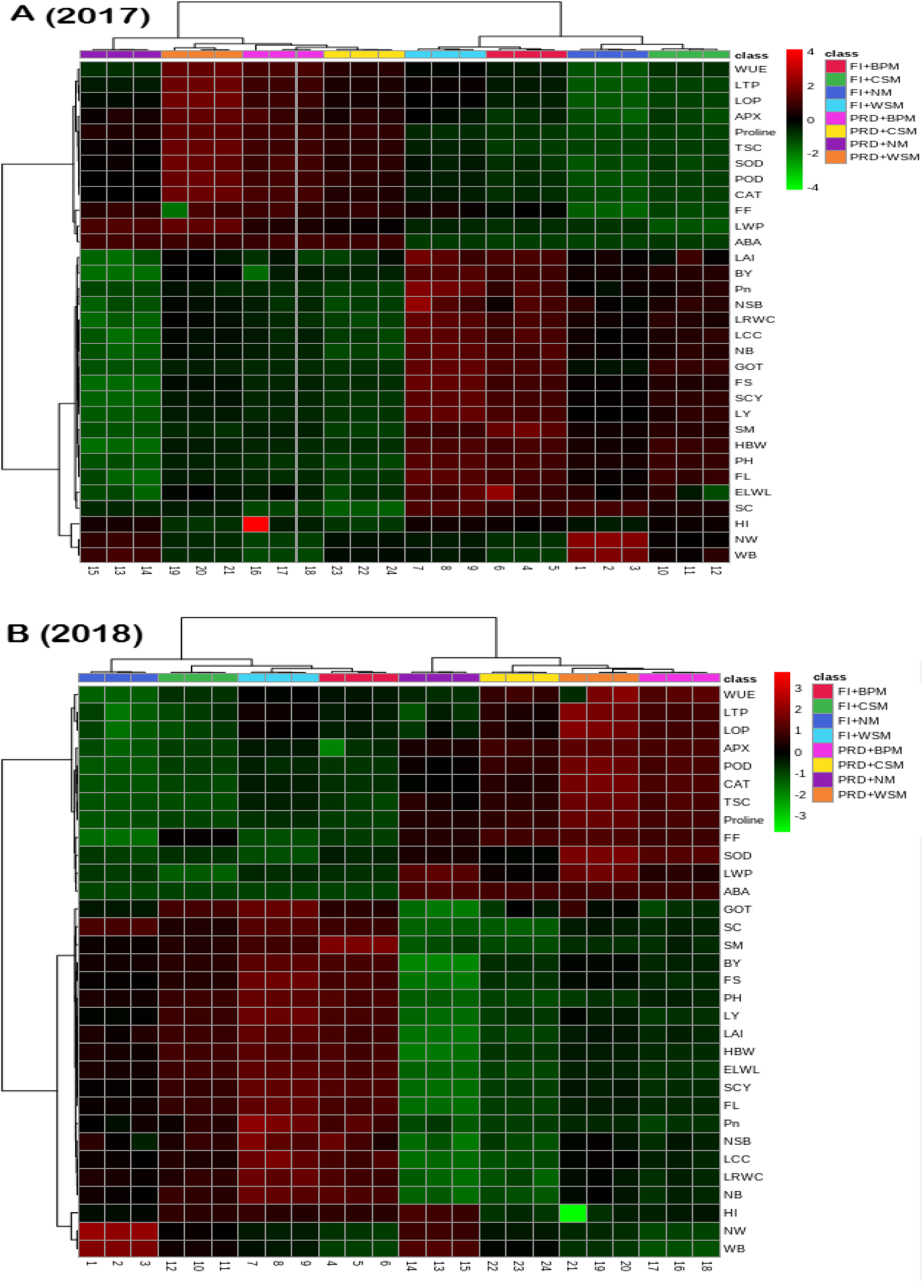
Heat map analysis with clustering and different classes according to the treatments devised the completed data from the range of 3 to −3 for easy and clear understanding of attributes for the year 2017 (A) and 2018 (B)

**Fig. 3:**
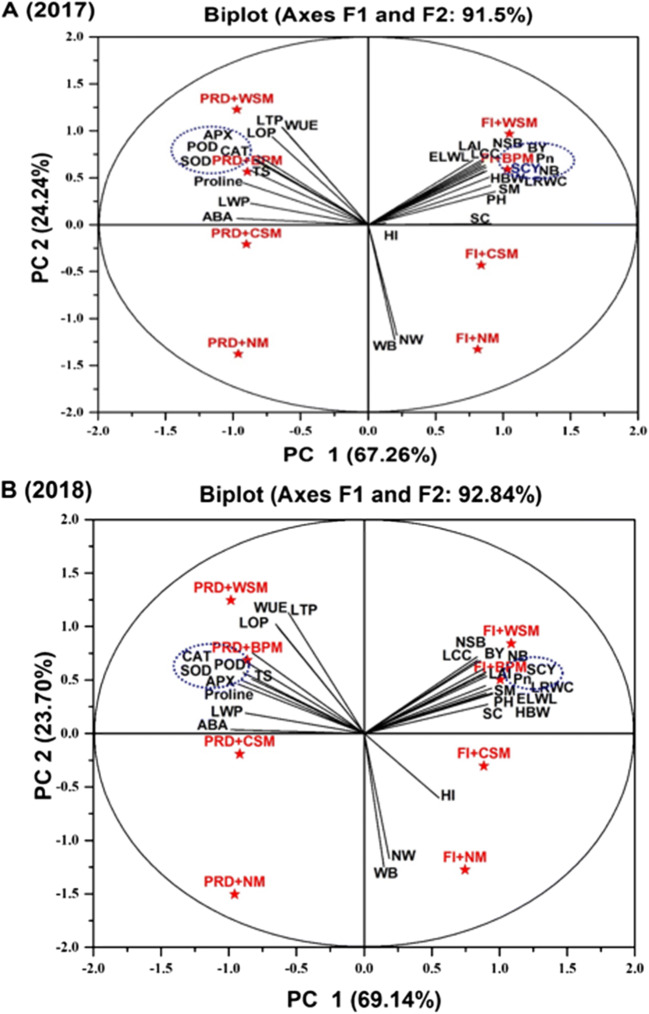
PCA analysis showing 91.5%and 92.84% total variability in the data in biplot graph for the year 2017 and 2018 respectively under PRD and mulches regimes. The angle cosine among two trait vectors indicates the correlation between traits, obtuse and acute angles indicate a negative and positive association, respectively, while the right angle between two vectors of variables indicates no correlation between them

Heat maps analyze the data into eight classes according to the treatment and then provided an instant view for a clear understanding of the traits by providing data range from 3 to −3 (Fig. 2). The PCA analysis demonstrated a distinct separation between PC1 and PC2 explaining total variations 91.5% and 92.82% for the year 2017 and 2018 respectively, among Pn, osmolytes, antioxidants, water-related traits, yield, and its contributing attributes. The angle cosine among two trait vectors indicates a correlation between traits, obtuse and acute angles indicate a negative and positive association, respectively, while the right angle between two vectors of variables indicates no correlation between them. The biplot graph from PCA analysis disclosed the many important associations for both years such as the positive correlation of SCY with its contributing attributes and many other important traits including Pn, LAI, PH, and LCC. Moreover, the biplot graph also explains the more vulnerable traits according to the treatment such as weed biomass and several weeds fall near FI+NM and antioxidants fall near PRD+BPM (Fig. 3).

## Discussion

### Effect of water and mulches regimes on yield and its contributing attributes along with quality traits of cotton

Climatic changes and pollution are depleting our water resources. Water is the main factor limiting crop yield and its low availability has harmful effects on the physiology, growth, and final yield of plants. In this scenario, partial rhizosphere drying (PRD) and mulches have some optimistic influences on the growth, development, and yield of various crops growing in water-limited areas. Plant height is a significant yield trait of cotton and low water availability severely reduced the cotton height (Iqbal et al. 2020; Rahman et al. 2016; Basal, 2010). We observed reduced cotton plant height under PRD for both years in the order, 2017 > 2018 accord with Iqbal and Raza (2019), Raza et al. (2017), and Stikic and Popovic (2003). However, WSM resulted in substantially better plant height among all mulches under PRD and more obvious in FI for both years in the order 2017 > 2018. During the early phases of cotton development, a low temperature in 2017 than in 2018 could be the reason for year-wise cotton height variation (Table 4). The reduced cotton plants height may correspond to the low turgidity in cells and their division under moisture stress conditions (Farooq and Basra, 2008), and improved cotton plants height responses to mulches reflect the sunlight, reduce the evaporation losses, and hence increase the moisture content of the soil in comparison to barren soil (Ahmad et al. 2015; Iqbal et al. 2020). The more pronounced increment in plant height in response to the WSM might be due to the quick and easy biodegradation of wheat straw, which may improve soil health and nutrient availability (Ahmad et al. 2020).

Leaf area index (LAI) is a very important factor in crop production. Drought stress reduced the leaf expansion and also disturbs the photosynthesis process ( Pettigrew, 2004). A low number of leaves capture less energy for glucose formation (Alves and Setter, 2004). The PRD-treated plants had less leaf area in comparison to FI due to less cell division and growth for both years in the sequence of 2018 > 2017. The same findings of the leaf area index were also found by Raza et al. (2017), Stikic and Popovic (2003), and Wang and De Kroon (2005). Different researches also showed less leaf area index in drought-stressed plants (Iqbal and Raza, 2019; Ihsanullah, 2009; Parida and Dagaonkar, 2007). Wheat straw mulch performed the best than other mulches for LAI in order of WSM>BPM>CSM>NM due to more availability of soil nutrients (Table 4).

The number of bolls per plant is one of the significant yield factors to drought stress in cotton plants. Drought stress mostly affects the boll formation and retention in cotton crops and the trend for both years was in the order of FI>PRD in accord to Ihsanullah (2009) and Pettigrew (2004). The higher number of bolls under mulch treatments was due to the high water retention and lower weed density, which provided a favorable condition for plant growth in an order of WSM>BPM>CSM>NM, as compared to non-mulch treatment. The higher temperature in 2018 also reduced the bolls retention in comparison to 2017. The number of sympodial branches per plant is also an important yield determinant of cotton. More branches mean more bolls and ultimately higher yield. The FI applied treatment attained more branches as compared to PRD. A higher number of branches under mulch treatments was due to more water conservation and less number of weeds thus provides a favorable condition for plant growth compared to un-mulched treatment (Ahmad et al. 2015; Ahmad et al. 2020). Treatments covered with wheat straw mulch produced more sympodial branches as compared to no mulch treatment and all other mulches were in a sequence of BPM>CSM>NM for both years in FI > PRD (Table 4).

Boll weight plays a key role in increasing seed cotton yield. The PRD applied treatments got less value for boll weight of cotton as compared to FI. Basal (2010) reported a decrease in bolls weight with decreasing the amount of water applied and with increasing temperature range in a sequence of 2017>2018. Treatments in which wheat straw mulch was used produced more bolls weight than BPM>CSM>NM (Table 4). More value of boll weight in mulched treatment is mainly due to the more retention/conservation of soil moisture which in turn helped in more photosynthetic rate and ultimately higher assimilates partitioning as compared to un-mulched treatment (Ahmad et al. 2015; Nasrullah and Khan, 2011).

The yield of the cotton plant is influenced by boll number and weight, number of seeds per boll, and fiber quantity per seed. These yield attributes are mainly dependent on growth and physiological processes. Low availability of water can disturb all the growth, physiology, metabolic, yield attributes, and finally the fiber quality in cotton crop. The FI achieved higher cottonseed than PRD for both years, which is in line with Ihsanullah (2009) and Ghaderi et al. (2012). The trend of cotton seed yield for both years in FI and PRD for mulches was WSM>BPM>CSM>NM but FI > PRD (Table 4). More seed cotton yield in mulch treatments was due to more moisture retention in soil, improved growth, more photosynthetic rate, and ultimately the higher yield attributes about non-mulched treatment. Schahbazian and Iran-Nejad (2006) and Yuan and Wu (2006) reported higher seed cotton yield under polythene mulch and straw mulch compared to NM.

Drought stress reduced the biological yield (BY) of cotton by reducing every growth-related parameter and ultimately the final yield. The trend of BY was PRD <FI. Similar findings were proposed by Ihsanullah (2009) and Ghaderi et al. (2012) who noted less value of biological yield in drought applied treatment. Wheat straw mulch produced a higher value of biological yield about BPM > CSM > NM. These observations are following the findings of Schahbazian and Iran-Nejad (2006) and Yuan and Wu Qun (2006). Weeds cause more damage to the crop plants and the final economic yield of various crops as compared to insect pests and different diseases. In various researches, it is proved that after 30–40 days of germination of cotton if proper weed control measures are not adopted yield may reduce up to 20–40%, and in the severe competition of weeds, it may lower the yield to 80% (Karlen et al*.*, 2002). The NM resulted in maximum weed density and biomass than CSM < WSM < BPM for both FI and PRD (Table 4) because mulch materials reflect the sunlight and in this way light cannot efficiently penetrate the mulch surface. As a consequence, weeds are not able for photosynthesis and cannot survive accord to Ather et al. (2013) and Nalayini (2009).

Ginning out turn (GOT %) is also called lint percent. The ultimate objective of cotton cultivation is lint production. FI got more GOT as compared to PRD-treated plants. Sahito et al. (2015) concluded that with increasing the irrigation frequency GOT was also increased in cotton crop. The same findings were proposed by Matthew et al. (2014). Among the various mulches used in the experiment maximum, GOT (lint percentage) was recorded in WSM about NM. Fiber length is also called staple length. The fiber length of a variety is an important quality character as it plays an important role in the textile industry. FI plants have more fiber length than PRD. Observations of the study for fiber length are following the results of Sahito et al. (2015), Matthew et al. (2014), and Ahmad et al. (2013). They conclude that with the reduction of moisture gradient in the soil the fiber length also reduced (Shareef et al. 2018b). Among the various mulches application, the wheat straw mulch attained the maximum fiber/staple length more in relation to NM (Table 5). Mulches can reduce the evaporation losses and increase soil moisture content (Iqbal and Raza, 2019; Ahmad et al. 2015; Nasrullah and Khan, 2011).

Fiber strength is an imperative fiber character that affects the spinning of the lint and the quality of the spun yarn. High fiber strength is the most desirable characteristic in the cotton crop. The trend of fiber strength was in sequence of PRD <FI (Table 5). Fiber strength is mostly dependent on the genetic makeup of genotypes and also on the moisture content available to the plants. More moisture available to the plants more will be the fiber strength (Sahito et al. 2015; Matthew et al. 2014; Ahmad et al. 2013; Shareef et al. 2018b). Application of different mulches got more fiber strength about NM. Maximum fiber strength was attained in WSM. Fiber fineness of various cotton varieties has soft and silky type on the other hand various genotypes have harsh and coarse fiber. PRD-treated cotton plants attained more fiber fineness than FI plants. Fiber fineness also depends on the genetic character of various varieties Sahito et al. (2015). Among the different mulches application, there was a non-significant effect on fiber fineness of cotton crop during the first year but it was found statistically highly significant during the second year that is maybe due to variation in temperature and moisture gradient.

### Effect of water and mulches regimes on water-related traits of cotton

Leaf water stress can be measured through excised leaf water loss (ELWL) and the water potential of the leaf. FI attained more ELWL as compared to PRD. NM treatment computed less excised leaf water loss as compared to wheat straw which performed mostly the best than other mulches. Tanvir and Sana-ullah (2006) counted similar findings of reduction in excised leaf water loss of cotton leaves under drought stress treatment (Iqbal and Raza, 2019). Water amount in plant leaf is mostly calculated by relative water content (RWC). PRD got less relative water in leaves in relation to FI (Table 5). PRD-treated plants have less cell division so their leaves are smaller than FI plants (Iqbal and Raza, 2019; Stikic and Popovic, 2003). Mulches performed the best at both FI and PRD than NM. The main reason behind this the mulches cut down the evaporation losses from the soil surface and hence improved the soil moisture to an optimum level (Iqbal and Raza, 2019; Iqbal et al. 2020; Ahmad et al. 2015).

Leaf water potential (LWP) is commonly used to calculate the potential energy of water in plant leaf tissues. It changes with a slight change in leaf osmotic potential. PRD has more LWP than FI for both years. Similar findings were also observed by Raza et al. (2017) and Wakrim et al. (2005). They recorded more negative values of leaf water potential about FI (Iqbal and Raza, 2019). The sequence of LWP for mulches was in the order of WSM>NM>BPM>CSM. Leaf osmotic potential represents the concentration of osmolites or salts in the leaf. Drought harms the osmotic potential value (Iqbal and Raza, 2019; Saleh, 2012). FI applied treatment achieved less negative value as compared to PRD treated plants (Table 5). More concentrations of these salts in stress situation is mainly due to the breakdown of larger molecules into smaller ones hence the maximum value of osmotic potential attains (Iqbal and Raza, 2019; Raza et al. 2017; Chutia and Borah, 2012). Using various mulch materials, more leaf osmotic potential value was recorded in WSM that was more than the NM plants.

Leaf turgor potential helps to improve the physiological activities going on in the leaves (Raza and Saleem, 2014). It is mostly dependent on the relative water content of the leaf. It is also known as turgor pressure. The PRD irrigated treatment showed higher leaf turgor potential as compared to FI. Among the various mulch treatments, more value of leaf turgor potential was observed in WSM that was more than the NM. Soil moisture percentage is the indication of moisture present in the soil. It varies with the type of soil, the climatic situation of an area, and also the delta of water for a crop. The PRD got less value of soil moisture than FI for both years in order of 2017 > 2018 (due to higher temperature). This large fluctuation is due to the reason that in PRD we conserved 50% of water by alternating the furrow irrigation. For mulch treatments, a higher percentage of soil moisture was recorded in BPM that was more than other mulches in order of WSM>CSM>NM for both the years (Table 5). Mulch materials made favorable conditions for cotton plants by conserving the water status in soil by reducing of weeds population that else use and transpire sufficient amount of water in field conditions (Iqbal et al. 2020; Iqbal and Andersen, 2019; Ahmad et al. 2015; Nasrullah and Khan, 2011).

Water use efficiency (WUE) is the ratio of dry matter production to the total amount of water applied. High values of WUE indicate an increased biomass production per unit of water used. WUE is an imperative attribute to compute the drought tolerance of crop species. PRD got more WUE as compared to FI. A higher value of WUE in mulched treatment was due to few evaporation losses and more water conservation. Treatments covered with wheat straw mulch showed 30.62% (2017) and 24.43% (2018) higher WUE compared to NM. In the first growing season, the temperature was more feasible in accord with Ihsanullah (2009) and Iqbal and Andersen (2019).

### Effect of water and mulches regimes on physio-biochemical traits of cotton

Drought stress is the main factor that affects the leaf chlorophyll content in crop plants (Iqbal and Raza, 2019; Hakam and DeEll, 2000: Ashrafuzzaman and Khan, 2000). The chlorophyll content is an excellent indicator of photosynthetic rate (Zhang et al. 2007). The PRD plants got less chlorophyll than the FI (Table 6). Raza et al. (2017) got less chlorophyll content in wheat cultivars during PRD in relation to FI (Iqbal and Andersen, 2019). Drought stress disturbs the activity of the enzyme (chlorophyllase), pigments in leaf mainly involved in the chlorophyll formation and hence the photosynthesis. Wheat straw mulch got the maximum chlorophyll content in comparison to all other mulches. Mulching positively affected cotton growth (Nasrullah and Khan, 2011) with a high content of chlorophyll in leaves (Iqbal and Raza, 2019).

The ecological interaction of plants and their surrounding is mainly due to Stomata. Crop response is also evaluated with the help of stomata conductance grown in water-limited areas (Iqbal and Raza, 2019). PRD plants got low stomata resistance than the FI plants. ABA production in PRD-treated plants is the main agent for the regulation of stomatal conductance for water-saving of plants otherwise it transpires from the plant body (Shareef and Gui, 2018; Tang et al., 2005; Du et al. 2008; Shareef et al. 2018a; Iqbal et al. 2020). However, stomata conductance value differs between PRD and FI plants but there is no significant effect on the photosynthetic rate or final yield of crop plants (Ahmadi et al. 2010). All the mulches performed excellent than the NM that got 2.12% (2017) less stomata conductance but during the second year (2018), CSM got 13.34% less stomatal conductance than WSM (Table 6).

The rate of photosynthesis and stomata resistance are mostly affected due to severe water stress in plants (Shareef and Gui, 2018; Shareef et al. 2018a; Iqbal et al. 2020). Under drought stress, the carboxylation sites of leaves get less carbon dioxide and consequently a low rate of photosynthesis. FI has higher values of photosynthesis than PRD for both the years in order of 2017 > 2018. All the mulches had positive effects on both the irrigation levels than NM. The sequence of photosynthesis in mulching was WSM > BPM > CSM > NM (Table 6). This higher rate of photosynthesis is primarily linked to the water conservation strategies in cotton i.e., PRD and mulching (Iqbal et al. 2020).

Soluble sugar content (SSC) has the primary role of osmoprotectants in various crop species under drought stress situations globally (Shareef and Gui, 2018; Shareef et al. 2018a; Iqbal and Raza, 2019). Most of the cell structures and organelles are protected and stabilized by these sugar contents. It can also maintain the turgor potential in plant leaves. The sequence of SSC is PRD > FI following Raza et al. (2017) and Iqbal and Raza (2019). Wheat straw mulch with PRD irrigation got more SSC than other mulches used in the experiment. More production of ABA (abscisic acid) occurs in the dry portion of roots that via xylem vessels reach to shoots and plant leaves (Davies and Zhang, 1991) where it partially closes the stomata aperture and in this way maintain the water status in plants under PRD (Shareef and Gui, 2018; Bauerle et al. 2006; Kang and Zhang, 2004; Liu et al. 2006; Liu et al. 2005; Shareef et al. 2018a). The PRD-applied treatment attained more leaf abscisic acid than the FI (Iqbal and Raza, 2019). All the mulches had the same effect on abscisic acid concentration in cotton leaves (Table 6).

Proline is commonly used as an osmolyte in osmotic adjustment in plants under stress conditions (Iqbal and Raza, 2019; Azooz et al. 2004). In drought stress situations, proline formation is commonly due to the conversion of other amino acids into proline and simultaneously inhibition of protein synthesis with protein break down into proline (Izanloo et al. 2008; Chiatante and Iorio, 2000). It is also helpful in osmotic adjustment in plants growing in water-deficient conditions. PRD attained more proline value in comparison to FI due to elevated concentration of ABA in PRD. More ABA initiates the gene (*P5CS*) expression that is the main cause of more proline concentrations (Ashraf and Foolad, 2007). Findings of the current research have similarity with Raza et al. (2017), Stikic and Popovic (2003), Khan et al. (2012), and Iqbal and Raza, (2019) who recorded more proline production in PRD plants about fully irrigated plants (Shareef and Gui, 2018; Shareef et al. 2018a).WSM with PRD irrigation attained more proline than other mulched treatments.

Reactive oxygen species (ROS) are mostly produced in abiotic stress (salt, water, heavy metals) conditions and sometimes during cold stress (Hu and Wu, 2010; Mutlu et al. 2009a, b). These have detrimental effects on various proteins and the structure of DNA in plant tissues (Kekeç et al. 2013; Bertrand et al. 2011). An efficient antioxidant (SOD, POD, CAT, APXetc) defense mechanism is indispensable for the continued existence of cell and organelles. Various antioxidant enzymes such as SOD, POD, CAT, and APX can reduce down the ROS by engulfing them or converting them into water molecule by various reactions which otherwise may be lethal for cells (Edreva, 2005; Rout and Shaw, 2001; Chaves and Maroco, 2003; Iqbal and Raza, 2019). Superoxide ions catalyzed in the presence of SOD into H_2_O_2_and O_2_. Then, CAT and APX convert this H_2_O_2_ into a water (H_2_O) molecule that can be available for plants in stress conditions due to this defense mechanism. When there is a large quantity of H_2_O_2_ then POD is activated for engulfing them and also to help for cell expansion. Higher activity of these antioxidants was recorded in PRD-treated plants about FI as these enzymes performed better for osmotic adjustment in water stress conditions (Iqbal and Raza, 2019). All the applied mulches in PRD treatments had also higher activity of these antioxidants (Table 6). The same findings (salt/drought vs. normal irrigation) in cotton were computed by Diego et al. (2003) and Ratnayak et al. (2003).

## Conclusions

This study explored PRD as an efficient irrigation technique along with WSM application under limited water supply, improving cotton yield and fiber quality under an arid climatic region. These improvements were accredited to many inter-connected physio-biochemical mechanisms such as enhanced water relations, photosynthesis, osmolyte accumulation, ABA production, and antioxidants activity to scavenge excess ROS production. PRD+WSM may prove as a good adaptation to grow better cotton under limited water as currently, the region is facing a water shortage. As PRD+WSM improves WUE and several biochemical processes which ultimately improved the yield and quality of the cotton crop, so it can be recommended for the cotton growers to adopt it as a potential adaptation strategy under current climatic scenarios in the region to achieve high cotton production and save water. Future studies may focus to elucidate the soil properties and microbial communities’ changes under WSM and especially evaluate the potential of these techniques under various climatic regions to evaluate the potential under future climate change seniors using modeling approaches.

## Data Availability

The datasets used and/or analyzed during the current study are available from the corresponding author on reasonable request.
